# Physiological and transcriptional immune responses of a non-model arthropod to infection with different entomopathogenic groups

**DOI:** 10.1371/journal.pone.0263620

**Published:** 2022-02-08

**Authors:** Joseph L. Black, Mason K. Clark, Gregory A. Sword

**Affiliations:** Department of Entomology, Texas A&M University, College Station, Texas, United States of America; University of California Riverside, UNITED STATES

## Abstract

Insect immune responses to multiple pathogen groups including viruses, bacteria, fungi, and entomopathogenic nematodes have traditionally been documented in model insects such as *Drosophila melanogaster*, or medically important insects such as *Aedes aegypti*. Despite their potential importance in understanding the efficacy of pathogens as biological control agents, these responses are infrequently studied in agriculturally important pests. Additionally, studies that investigate responses of a host species to different pathogen groups are uncommon, and typically focus on only a single time point during infection. As such, a robust understanding of immune system responses over the time of infection is often lacking in many pest species. This study was conducted to understand how 3^rd^ instar larvae of the major insect pest *Helicoverpa zea* responded through the course of an infection by four different pathogenic groups: viruses, bacteria, fungi, and entomopathogenic nematodes; by sampling at three different times post-inoculation. Physiological immune responses were assessed at 4-, 24-, and 48-hours post-infection by measuring hemolymph phenoloxidase concentrations, hemolymph prophenoloxidase concentrations, hemocyte counts, and encapsulation ability. Transcriptional immune responses were measured at 24-, 48-, and 72-hours post-infection by quantifying the expression of *PPO2*, *Argonaute-2*, *JNK*, *Dorsal*, and *Relish*. This gene set covers the major known immune pathways: phenoloxidase cascade, siRNA, JNK pathway, Toll pathway, and IMD pathway. Our results indicate *H*. *zea* has an extreme immune response to *Bacillus thuringiensis* bacteria, a mild response to *Helicoverpa armigera* nucleopolyhedrovirus, and little-to-no detectable response to either the fungus *Beauveria bassiana* or *Steinernema carpocapsae* nematodes.

## Introduction

The insect innate immune system is non-specific and assumed to be without memory, yet it can be vitally effective at attacking and overcoming challenges by pathogens or parasitoids [1–3]. Insect immune systems are able to recognize non-self and altered-self molecular structures by means of pathogen recognition receptors (PRRs) that bind to lipid particles and pathogen-associated molecular patterns (PAMPs). PAMPs include lipopolysaccharides (LPSs), peptidoglycans (PGNs), and glucans common to pathogens. The PRR-PAMP identification mechanism allows for a targeted immune response that has minimal direct impact on the uninfected portions of the host [4–8]. Cytokines and signaling pathways such as Toll, IMD, JNK and JAK/STAT are important in the identification of non-self/altered-self, and the initiation of an immune response [9–15]. The resulting immune response to invading pathogens is a two-pronged response consisting of humoral and cellular defenses. The humoral defense consists of antimicrobial peptide (AMP) production via the IMD and Toll signaling pathway, and the formation of other effector molecules through complex proteolytic cascades such as the phenoloxidase (PO) cascade (Reviewed in [15–19]). The second primary component of an insect’s innate immune system is the cellular response to infection involving encapsulation, nodulation, phagocytosis, and apoptosis of pathogens mediated by the JNK signaling pathway, hemocyte signaling, and regulation by eicosanoids (Reviewed in: [20–22]).

### Responses to specific pathogen groups

Insect defense mechanisms against viral pathogens utilize several different immune pathways but are relatively limited compared to other pathogen responses. There are many families of viruses that are pathogenic to insects; however, baculoviruses are the most widely studied and best understood. As such, they are currently being used in commercial agricultural production as biopesticides. Baculoviruses are large viruses with a circular dsDNA genome. Most baculoviruses are highly host specific and only capable of replicating in a narrow range of related hosts. Baculoviruses gain entry into the host hemocoel through ingestion or wounds in order to initiate cellular invasion and replication. If ingested, the primary infection is in the midgut epithelial cells, and within 2 hours post-infection (hpi), a secondary infection can be established in the tracheal system [23]. Once the infection is established in the trachea, the infection moves to the hemocoel and becomes systemic. Cellular and humoral defense mechanisms, such as nodule formation, phagocytosis, and production of PO-derived reactive oxygen species, have been observed as potential antiviral defense mechanisms; however, the primary antiviral defense appears to be RNA interference (RNAi) [24–30]. Currently, there are four types of RNAs implicated in host defense by RNAi: small-interfering RNA (siRNA), microRNA (miRNA), piwi-RNA (piRNA), and long none-coding RNA (lncRNA). Of these, the siRNA pathway has been identified as the more potent antiviral defense mechanism during infection [24, 31–35]. Internalization of exogenous viral dsRNA activates the siRNA pathway for RNA degradation [36]. Upon internalization, viral dsRNA is bound to the heterodimer Dicer-2/R2D2 complex for ribonuclease activity [36]. Dicer-2 processes the dsRNA into 21-nt siRNA duplexes [37, 38]. These duplexes are then loaded and unwound onto a pre-RISC complex where the unstable passenger strand is discarded [36]. The remaining guide strand loaded onto the RISC complex is used by Argonaute-2 (AGO2) as the template strand to degrade sequence specific strands of viral RNA through cleavage [39]. Although this RNA-specific defense mechanism has been shown to be effective against (-) RNA viruses, (+) RNA viruses, and dsRNA viruses, recent research has demonstrated that DNA viruses are also targets of the antiviral RNAi response [29, 31, 33–35, 40–42].

Like viruses, bacterial entomopathogens must gain entry to the host either through oral ingestion or through orifices, such as spiracles or wounds. Once the physical barriers have been surmounted, the insect host relies on cellular and humoral immune responses to clear the infection. Once PRRs recognize and bind to the PAMPs produced by the bacterial pathogen, an immune response is elicited [43–45]. Cellular responses to bacterial invasion include phagocytosis and nodule formation. Humoral responses consist of the PO cascade and AMP production via the activation of either the Toll or IMD pathway depending on the type of the LPSs in the bacterial cell wall [21, 46–50].

Fungal entomopathogens are capable of directly penetrating the host cuticle through the deployment of an appressorium, or invade through orifices such as spiracles, wounds, or oral ingestion [51]. Once the infection reaches the hemocoel, hemocytes will begin encapsulating spores, and the PO cascade melanizes the capsule, fumigating it with reactive oxygen species [48, 52–54]. Furthermore, the major fungal cell wall component β-1,3-glucan functions in insects as a PAMP that will induce the activation of the Toll pathway, producing AMPs with activity against fungi [25, 47, 55].

Entomopathogenic nematodes are also capable of penetrating the insect cuticle or invade orifices similarly to fungal pathogens. However, recent evidence suggests that the cuticle of some nematodes does not elicit an immune response from the insect host, thereby evading detection until farther into the infection [56–60]. During nematode infections, the primary immune response is one of encapsulation and melanization [22]. Once the nematode’s bacterial symbiont is detected, the host will respond with AMP production based on the signaling pathway activated [22, 59].

### Expanding immune studies to non-model insects

Currently, we have a fairly robust understanding of insect developmental signaling pathways and how duplication and modification of those pathways has given rise to complex immune signaling pathways [61, 62]. We also understand the basic mechanisms by which these signaling pathways are triggered, and the effector molecules they produce. However, most of the established immune pathway-pathogen associations have been studied in model organisms, such as *Drosophila melanogaster*, and only recently has research expanded to include broader insect taxa. The increasing availability of genomic data along with tools to study gene expression, such as real time quantitative polymerase chain reaction (qPCR), provides new opportunities to expand our understanding of insect immune responses in a range of socially or economically important insects, either for their control or conservation. Currently, hypothesis-driven immune-related studies either continue to employ physiological analyses or utilize transcriptional assays to measure immune responses, but it is uncommon to find both analyses implemented [63, 64]. Experiments commonly focus on the immune response to a single pathogen, sampling at one or more times during the infection. Or experiments explore the immune response to multiple pathogens, but focus on a single point during the infection [63].

We set out to broaden our knowledge of immunity in economically important non-model insects by conducting a systematic assessment of multiple insect defensive responses over the course of infection when challenged by several major pathogen groups. In this study, we utilized *Helicoverpa zea* larvae, which is one of the most important agricultural pests in the Western Hemisphere [65–69]. These larvae were separately exposed to four different pathogens, and immune responses were measured via physiological and transcriptional analyses at three different points across the infection cycle. Therefore, our objective was to determine how *H*. *zea* immune system responds to different pathogens throughout the infection process. We hypothesize that the immune response of *H*. *zea* will mirror the responses reported in model insects and other closely related species. This study provides a unique perspective of how a highly destructive pest copes with invasion by a broad range of different pathogens. Furthermore, this study provides the foundation for future studies investigating the ecology and evolution of pathogen resistance in *Helicoverpa* species.

## Materials and methods

### Insects and pathogens

*Helicoverpa zea* caterpillars were purchased from Benzon Research Inc. (Carlisle, PA) as eggs and were reared on artificial diet purchased from Southland Products Inc. (Lake Village, AR) until reaching the targeted instar. Larvae were maintained in rearing chambers under a constant temperature of 25°C, relative humidity of 70%, and light-dark ratio of 14:10 for all experiments. The strain of *Helicoverpa armigera* nucleopolyhedrovirus (HearNPV) was provided by AgBiTech LLC (Fort Worth, TX), and is listed under the trade name Heligen®. To get to the desired concentration for inoculation, the highly concentrated viral solution was serially diluted from 7.5 × 10^9^ occlusion bodies/mL to 7.5×10^5^ occlusion bodies/mL based on previous research (Black et al. unpublished). The *Beauveria bassiana* strain GHA was isolated from BotaniGard Maxx® purchased from BioWorks Inc. (Victor, NY). The spores were extracted by placing 30mL of the solution into a 50mL Falcon tube and centrifuging at 3000 rpm for 10 minutes, then removing the supernatant and adding 30mL of sterile water. The solution was then vortexed, and this process was repeated three times before the final pellet was resuspended in sterile water and stored at 4°C until needed. Spore suspension viability was tested prior to use by making a serial dilution and plating the 5^th^ and 6^th^ dilution. The plates were allowed to incubate at room temperature for three days and then colonies were counted and multiplied by the dilution factor to determine viable spore concentration in the spore suspension. The *Bacillus thuringiensis* pathogen was diluted from Thuricide BT® purchased from Southern AG Insecticides, Inc. (Hendersonville, NC) to 7.5×10^5^ CFUs/mL. *Steinernema carpocapsae* was purchased from ARBICO Organics (Oro Valley, AZ), and serially diluted to 7.5×10^5^ nematodes/mL.

### Inoculation procedures

Once *H*. *zea* larvae molted to 3^rd^ instar, they were inoculated with one of five treatments determined prior to initiation of the experiment. This was done by pipetting a 10μL drop of liquid containing either the treatment pathogen dosage or a control of sterile deionized water onto a fresh piece of artificial diet, approximately 50mg, where it was absorbed. Inoculation time zero was defined as this point of pathogen introduction into the individual larva’s environment, which was a sterilized 2oz deli cup (ULINE, Pleasant Prairie, WI). The larvae were then allowed to feed on the infested diet and only those that consumed the inoculated diet cube were utilized in each of the two experiments described below. Although oral infection is not the primary infection route for entomopathogenic fungi or nematodes, they are still capable of infecting through the midgut [69, 70]. Also, the surface contamination of the diet should not inhibit the potential for infection through the insect cuticle or other orifices, but would simulate a more likely environment when used as a foliar bio-pesticide [71].

### Experiment 1: Temporal physiological immune response

This experiment utilized five pathogen treatments (Control, HearNPV, *B*. *bassiana*, *B*. *thuringiensis*, and *S*. *carpocapsae*), and subdivided each treatment into sample times. Three temporal sampling points of 4 hours, 24 hours, and 48 hours post-inoculation were implemented to develop an understanding of how the *H*. *zea* immune response changes during a pathogen invasion. Later temporal sample points were not possible because most larvae succumbed to the pathogens by three days and survivors across all pathogens were too few for meaningful analysis. Individual larvae were sampled by extracting their hemolymph at the designated sample times as described below. Hemolymph from two larvae were pooled to make one biological replicate. Each pathogen × time-treatment had 32–34 biological replicates collected across three independent trials.

### Hemolymph extraction

Hemolymph extraction occurred by sterilizing the larva with an ethanol wash, weighing the larva, and then chilling the larva on ice before piercing the larva with a sterile 27-gauge needle between the second pair of prolegs. The insect hemolymph was allowed to drain directly into an Eppendorf tube on ice and placed into a -20°C freezer immediately upon completion of the extraction. While extracted volumes varied across biological samples, each sample had at least 70μL to complete all the physiological assays described below.

### Hemolymph phenoloxidase and prophenoloxidase assay

An 8μL aliquot of hemolymph was added to 360μL of sodium cacodylate (NaCac) in a 2mL microcentrifuge tube. The sample was then evenly divided into two 2mL microcentrifuge tubes. One tube had the prophenoloxidase (PPO) activated by adding 20μL of 20mg/mL chymotrypsin suspended in NaCac buffer, while the other tube served as the spontaneously activated phenoloxidase (PO) control with 20μL NaCac added. Samples were incubated at 25°C for one hour to allow the PPO time to be activated prior to microplate reader analysis. All analyses were run in duplicate using Costar® 96 well flat bottom plates and analyzed in an Infinite M200 Pro microplate reader (Tecan, Mӓnnedorf, Switzerland). Plates were first loaded with 90μL of the sample solution per well, and then 90μL of 4mM dopamine was pipetted into each well. Once all wells had both the sample solution and dopamine, the plate was placed into the microplate reader and the absorbance was measured at 492nm. The amount of phenoloxidase in the sample was calculated in phenoloxidase units, where one unit is the amount of enzyme required to increase the absorbance by 0.001 per minute.

### Hemolymph protein assay

Protein was measured using a BCA Protein Assay Kit II (BioVision Inc., Milpitas, CA) by adding 25μL of the hemolymph solution to 200μL of the BCA working reagent in each of the Costar® 96 well flat bottom plate wells. The plate was covered and incubated at 37°C for 30 minutes. After the incubation the absorbance was measured at 562nm. A standard curve using the provided standards was utilized to determine protein concentration (μg/mL). Once protein concentrations were known, phenoloxidase units were expressed as phenoloxidase units per mg of protein.

### Hemocyte count

Hemocyte counts were determined using an improved Neubauer hemocytometer. The hemocytometer was loaded with 8μL of pure hemolymph, allowed to settle for 20 minutes and the five non-adjacent squares were counted on each side of the hemocytometer to give an estimate of hemocyte density.

### Antimicrobial activity assay

Lytic activity against the bacterium *Micrococcus lysodeikticus* was determined using a lytic zone assay. Agar plates were made prior to the assay by mixing 10mL of agar suspension containing the following: 1.5g agar, 0.75g *M*. *lysodeikticus* in 50mL 0.2 M potassium phosphate buffer, 0.1mg/mL streptomycin sulphate, and 67mM potassium phosphate buffer (pH 6.4) and pouring the mixture into a plastic petri dish and stored in a 4°C refrigerator. For each plate, approximately 13 holes with a diameter of 2mm were punched into the agar and filled with 1μL of hemolymph, with two technical replicates per sample. The plates were incubated at 32˚C for 24 hours, photographed, and the diameter of the clear zones calculated with ImageJ imaging software. Standard curves were obtained using a serial dilution of egg white lysozyme, and concentration of egg white lysozyme equivalents were calculated. Standard curves were developed for each batch of plates. Based on the logarithmic connection to lysozyme concentration, diameters of lytic zones obtained from the hemolymph samples were converted to HLAs (ng/μL–equivalents of hen egg white lysozyme activity).

### Encapsulation response assay

Immediately after the hemolymph extraction, a 3mm long piece of nylon monofilament was inserted completely into the puncture wound of each larva in such a way to minimize the potential of rupturing the midgut. Surviving larvae were returned to diet for 24 hours. After that time, the surviving larvae were frozen and upon death the nylon monofilament was dissected out, mounted on a slide and photographed. The level of melanization and area of cell cover was quantified using ImageJ [72] imaging software distributed by Fiji [73]. One larva in each pooled biological sample was subjected to an encapsulation assay. If the gut was ruptured or the nylon filament was not recovered during the dissection, these larval samples were discarded.

### Statistical analysis

Pair-wise MANOVAs were conducted for each Pathogen × Control pairing, with Treatment and Time as main effects, and the measured immune responses as dependent variables. The average pooled larval weight was used as a covariate since all dependent variables were analyzed for all samples. Pillai’s trace statistic was used to compare differences from the Control. Then, each immune response was subjected to an ANOVA and Tukey’s HSD. All data were checked for conformity and normalcy. All analyses were conducted in R Studio [74].

### Experiment 2: Temporal transcriptional immune response

This experiment utilized the same five pathogen treatments as in Experiment 1, but each treatment was subdivided into three different sample times: 24 hours, 48 hours, and 72 hours post-inoculation. Changes in the expression of genes involved in the major immune pathways were measured as opposed to physiological immune responses. *Actin*, *RPS3*, *PPO2*, *Argonaute-2*, *JNK*, *Dorsal*, and *Relish* were the genes of interest. *Dorsal* and *Relish* were targeted rather than AMP transcripts because they are the transcription factors transcribing all the AMPs. Furthermore, there are many different AMPs between these two pathways, but by focusing on *Dorsal* and *Relish* we can still determine up-regulation or down-regulation of the immune pathway. Twenty-five larvae were reared for each pathogen × sample time treatment combination. Five larvae were pooled for each biological replicate, resulting in five biological replicates per pathogen x sample time treatment combination. Hemolymph was extracted as described above, except immediately following extraction, the 2mL microcentrifuge tubes were flash frozen in liquid nitrogen then stored in a -80°C freezer. RNA was extracted from the hemolymph samples using the RNeasy Mini Kit (Qiagen, Hilden, Germany). RNA concentrations were determined by using a NanoView Plus (General Electric, Boston, MA). RNA concentrations were then standardized to 100ng/μL before being converted to cDNA using iScript gDNA Clear cDNA Synthesis Kit (Bio-Rad Laboratories, Hercules, CA). The resulting DNA concentrations were determined with a NanoView Plus, and diluted to 100ng/μL by adding RNase and DNase free water. Once sample DNA concentrations were standardized, Quantitative Real-Time PCR (qPCR) was conducted using primers targeting specific immune genes, *Actin* as a housekeeping gene and *RPS3* as a verification gene that *Actin* was not differentially expressed across treatments (Table 1). Transcript-specific primers were designed by first extracting putative transcript sequences from the published *H*. *zea* draft genome and associated annotation file using gffread [75, 76]. This generated a sequence file of parsed mRNA and coding sequences (CDSes) from which we ran BLASTn searches using *Helicoverpa armigera* CDSes of *Actin*, *RPS3*, *PPO2*, *Argonaute-2*, *JNK*, *Dorsal*, and *Relish* as the query. The obtained *H*. *zea* transcripts were then secondarily validated through BLAST searches of the NCBI database to confirm sequence identifications. The obtained transcripts were passed through the PrimerQuest™ Tool provided by Integrated DNA Technologies to generate qPCR primers. Conventional PCR products were obtained from each primer pair and purified using a Monarch DNA Gel Extraction Kit (T1020S) and submitted for Sanger Sequencing to validate their specificity to the desired transcripts. qPCR was conducted using SYBR™ Green (Bio-Rad Laboratories, Hercules, CA) and Precision Blue Real-Time PCR Dye (Bio-Rad Laboratories, Hercules, CA) in a C1000 Touch Thermal Cycler with the CFX384 Real-Time System attachment (Bio-Rad Laboratories, Hercules, CA). Data was then exported into CFX Maestro (Bio-Rad Laboratories, Hercules, CA) software, and analyzed using ANOVA and Tukey’s HSD post hoc test in R Studio [74]. All target genes were previously determined to be differentially expressed during pathogenic infection in *H*. *armigera* and *S*. *frugiperda*, two species closely related to *H*. *zea* [48, 77].

**Table 1:** Forward and reverse primers used in the transcriptional analyses.

Gene of Interest		Primer Sequence	Annealing Temp. (°C)	Size (bp)
Actin	Forward	ATGGGACAGAAGGACTCGTA	54.9	100
	Reverse	GGTGCCAGATCTTCTCCATATC	54.8	
PPO2	Forward	GATTACTCCGAAGGGTGACAAA	54.6	785
	Reverse	ACGGTGAACTGAGGGTATCT	55.2	
JNK	Forward	GAATGTCGCCATCAAGAAGTTG	54.4	751
	Reverse	ACGCGTTTAGAAGACCGATTAT	54.1	
Dorsal	Forward	TGTCACCAAAGATGAGCCTTAC	54.9	543
	Reverse	CGAGGTTCTTGAACTGGTACTC	54.6	
Relish	Forward	TGTGATTGACTGTGCGTGATA	54.2	750
	Reverse	GGAGAACTATGAGGAGGAGAGT	54.9	
Argonaute-2	Forward	TCAGGGCCTACTCCTGTATT	54.9	107
	Reverse	GGTGGCATAGCAGTAGAAGTAG	54.8	
Ribosomal Protein S3	Forward	CGGCTGTCCAATAGGATCTTC	54.8	219
	Reverse	CAGCCTCTTCATCTCATCCTTG	54.9	

## Results

### Experiment 1: Physiological immune response

#### Viral entomopathogen: *Helicoverpa armigera* nucleopolyhedrovirus

**Fig 1:**
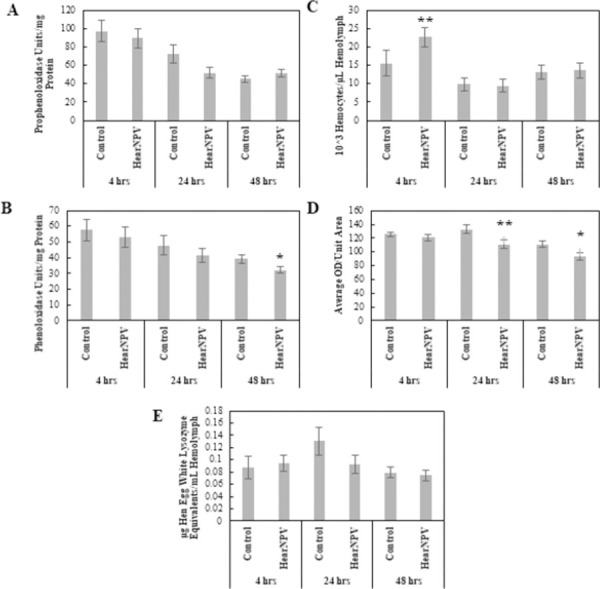
Comparison of physiological immune responses between control and *Helicoverpa armigera* nucleopolyhedrovirus-infected *Helicoverpa zea* over time. (A) Prophenoloxidase levels as a function of hemolymph protein levels (Mean ± SE), (B) Phenoloxidase levels as a function of hemolymph protein levels (Mean ± SE), (C) Total hemocyte numbers (×10^3^) per μl of hemolymph (Mean ± SE), (D) Encapsulation activity reported in ocular density per unit area (Mean ± SE), and (E) Lysozyme concentrations reported in hen egg white lysozyme equivalent per ml of hemolymph (Mean ± SE). A single asterisk indicates a marginally significant effect (ANOVA, P < 0.1) of treatment relative to the Control at that time point. Double asterisks indicate a stronger significant effect (ANOVA, P < 0.05) of treatment relative to the Control at that time point (n = 74–100 insects per treatment).

The physiological responses of prophenoloxidase (PPO) and phenoloxidase (PO) levels, lysozyme concentrations, number of hemocytes, and encapsulation ability for Control larvae and larvae infected with HearNPV were analyzed using a MANOVA with main effects being Treatment and Time. There was a significant Treatment effect (Pillai = 0.153, *F*_5, 143_ = 5.18, p < 0.001) and a significant Time effect (Pillai = 0.435, *F*_10, 288_ = 8.00, p < 0.001); however, the Treatment × Time interaction was not significant. Analysis of variance revealed there was a significant increase in the number of hemocytes in larvae infected with HearNPV compared to the Control at 4 hours post-inoculation (hpi) (α = 0.05, *F*_1, 65_ = 4.21, p = 0.0403), but all other physiological measurements were not significantly different from the Control group (Fig 1). By 24 hpi, only encapsulation ability was significantly lower for HearNPV-infected larvae (α = 0.1, *F*_1, 44_ = 3.94, p = 0.053), which was continued at 48 hpi (α = 0.1, *F*_1, 53_ = 3.95, p = 0.052) (Fig 1). Also, at 48 hpi, PO concentrations were significantly lower in HearNPV-infected larvae compared to the Control (α = 0.1, *F*_1, 66_ = 3.19, p = 0.079), while all other physiological responses were not significantly different from the Control (Fig 1).

#### Bacterial entomopathogen: *Bacillus thuringiensis*

**Fig 2:**
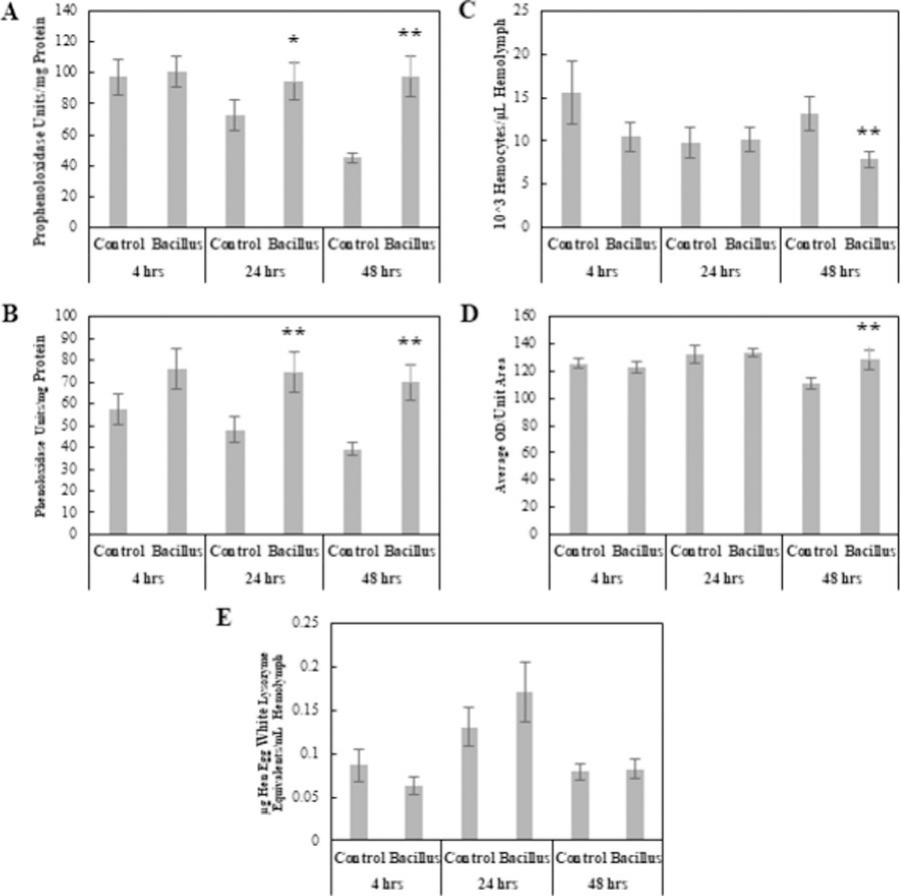
Comparison of physiological immune responses between control and *Bacillus thuringiensis*-infected *Helicoverpa zea*. (A) Prophenoloxidase levels as a function of hemolymph protein levels (Mean ± SE), (B) Phenoloxidase levels as a function of hemolymph protein levels (Mean ± SE), (C) Total hemocyte numbers (×10^3^) per μl of hemolymph (Mean ± SE), (D) Encapsulation activity reported in ocular density per unit area (Mean ± SE), and (E) Lysozyme concentrations reported in hen egg white lysozyme equivalent per ml of hemolymph (Mean ± SE). A single asterisk indicates a marginally significant effect (ANOVA, P < 0.1) of treatment relative to the Control at that time point. Double asterisks indicate a stronger significant effect (ANOVA, P < 0.05) of treatment relative to the Control at that time point (n = 60–99 insects per treatment).

The MANOVA results showed there was a significant Treatment × Time interaction (Pillai = 0.207, *F*_10, 260_ = 3.01, p < 0.001), and both Treatment and Time were independently significant. ANOVAs revealed there was no significant differences across all physiological responses measured at 4 hpi; however, by 24 hpi, PO and PPO levels had increased in *B*. *thuringiensis*-infected larvae compared to Control larvae (*F*_1, 62_ = 6.33, p < 0.05; *F*_1, 62_ = 3.19, p < 0.1) (Fig 2). At 48 hpi, encapsulation ability, PO levels, and PPO levels were significantly higher in *B*. *thuringiensis*-infected larvae compared to Control larvae (*F*_1, 47_ = 5.43, p <0.05; *F*_1, 66_ = 13.8, p < 0.001; *F*_1, 66_ = 15.4, p < 0.001), and hemocyte number was significantly lower in *B*. *thuringiensis*-infected larvae compared to Control larvae (*F*_1, 66_ = 8.63, p < 0.01) (Fig 2).

#### Fungal entomopathogen: *Beauveria bassiana*

**Fig 3:**
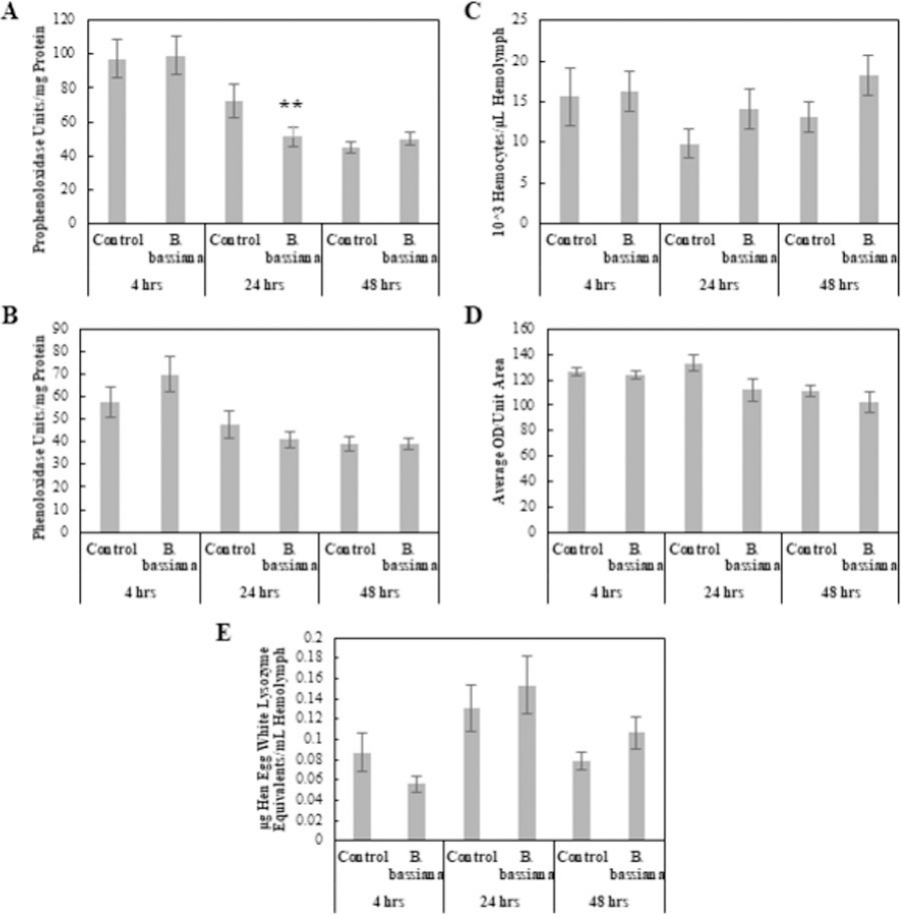
Comparison of physiological immune responses between control and *Beauveria bassiana*-infected *Helicoverpa zea*. (A) Prophenoloxidase levels as a function of hemolymph protein levels (Mean ± SE), (B) Phenoloxidase levels as a function of hemolymph protein levels (Mean ± SE), (C) Total hemocyte numbers (×10^3^) per μl of hemolymph (Mean ± SE), (D) Encapsulation activity reported in ocular density per unit area (Mean ± SE), and (E) Lysozyme concentrations reported in hen egg white lysozyme equivalent per ml of hemolymph (Mean ± SE). A single asterisk indicates a marginally significant effect (ANOVA, P < 0.1) of treatment relative to the Control at that time point. Double asterisks indicate a stronger significant effect (ANOVA, P < 0.05) of treatment relative to the Control at that time point (n = 62–100 insects per treatment).

MANOVA results comparing *B*. *bassiana*-infected larvae to Control larvae were not significant for Treatment or Treatment × Time interaction, but were significant for Time, showing that the physiological response changes over time, but that it is not necessarily an immune response change (Pillai = 0.342, *F*_10, 264_ = 5.45, p < 0.001). PPO levels in *B*. *bassiana*-infected larvae was significantly lower than Control larvae 24 hpi (*F*_1, 62_ = 2.97, p < 0.1) (Fig 3) and no other physiological immune response was significantly different from the Control.

#### Entomopathogenic nematode: *Steinernema carpocapsae*

**Fig 4:**
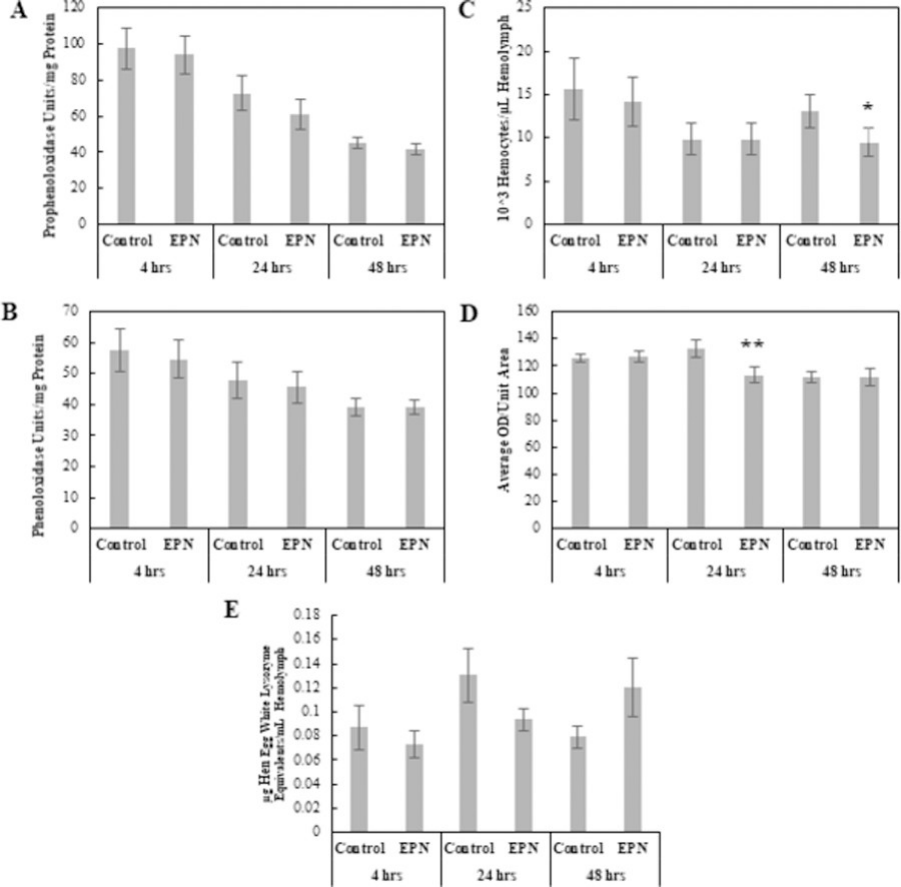
Comparison of physiological immune responses between control and *Steinernema carpocapsae*-infected *Helicoverpa zea*. (A) Prophenoloxidase levels as a function of hemolymph protein levels (Mean ± SE), (B) Phenoloxidase levels as a function of hemolymph protein levels (Mean ± SE), (C) Total hemocyte numbers (×10^3^) per μl of hemolymph (Mean ± SE), (D) Encapsulation activity reported in ocular density per unit area (Mean ± SE), and (E) Lysozyme concentrations reported in hen egg white lysozyme equivalent per ml of hemolymph (Mean ± SE). A single asterisk indicates a marginally significant effect (ANOVA, P < 0.1) of treatment relative to the Control at that time point. Double asterisks indicate a stronger significant effect (ANOVA, P < 0.05) of treatment relative to the Control at that time point (*n* = 70–99 insects per treatment).

The MANOVA results showed a significant Treatment × Time interaction effect (Pillai = 0.142, *F*_10, 280_ = 2.14, p < 0.05) and a significant Time effect (Pillai = 0.414, *F*_10, 280_ = 7.30, p < 0.001), but no significant effect by Treatment. ANOVAs revealed no significant differences in physiological responses between Control larvae and larvae infected with *S*. *carpocapsae* at 4 hpi. By 24 hpi, encapsulation ability was decreased for *S*. *carpocapsae*-infected larvae compared to Control larvae (*F*_1, 45_ = 3.59, p < 0.1) (Fig 4). At 48 hpi, hemocyte numbers were significantly lower in *S*. *carpocapsae*-infected larvae (*F*_1, 64_ = 3.32, p < 0.1) (Fig 4).

### Experiment 2: Temporal transcriptional immune response

#### Viral entomopathogen: *Helicoverpa armigera* nucleopolyhedrovirus

**Fig 5:**
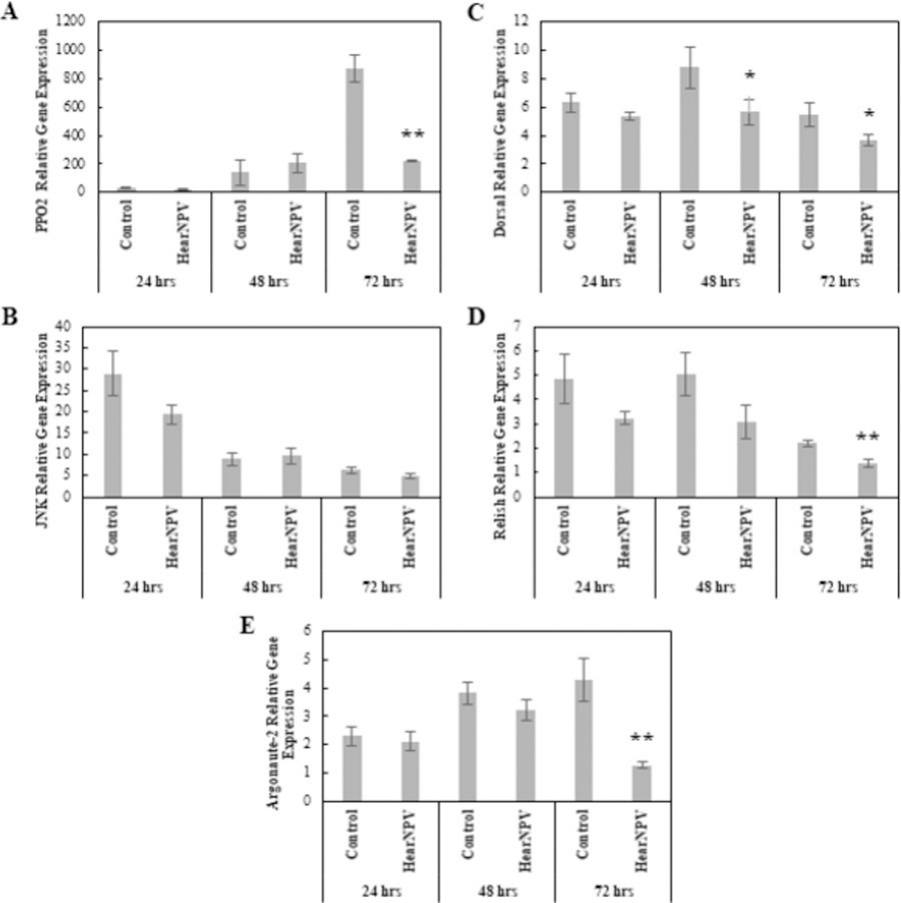
Comparison of relative expression levels of immune related genes in control and *Helicoverpa armigera* nucleopolyhedrovirus-infected *Helicoverpa zea*. (A) *PPO-2* (Mean ± SE), (B) *JNK* (Mean ± SE), (C) *Dorsal* (Mean ± SE), (D) *Relish* (Mean ± SE), and (E) *Argonaute-2* (Mean ± SE). *Actin* was used as the housekeeping gene. The single asterisk indicates a significant effect (ANOVA, *P* < 0.1) of treatment relative to the Control at that time point. The double asterisk indicates a stronger significant effect (ANOVA, *P* < 0.05) of treatment relative to the Control at that time point (*n* = 5 insects per treatment).

The relative gene expression levels for several different immune response signaling pathways were analyzed in a MANOVA with Treatment and Time as main effects, and relative gene expression levels of *Dorsal*, *Argonaute-2*, *PPO-2*, *JNK*, and *Relish* as variables. MANOVAs comparing HearNPV-infected larval gene expression to Control larval gene expression revealed a significant interaction effect of Treatment × Time (Pillai = 0.974, *F*_10, 42_ = 3.99, p < 0.001), and significant effects by both Treatment and Time independently (Pillai = 0.591, *F*_5, 20_ = 5.79, p < 0.001; Pillai = 1.49, *F*_10, 42_ = 12.26, p < 0.001). ANOVAs revealed no significant differences across immune gene expression levels 24 hpi. By 48 hpi, only *Dorsal* gene expression differed from the Control, with significantly lower expression levels in HearNPV-infected larvae (*F*_1,8_ = 3.15, p < 0.1) (Fig 5). At 72 hpi, *Argonaute-2*, *Dorsal*, *PPO-2*, and *Relish* genes were all differentially expressed compared to the Control, with significantly lower levels of expression (*F*_1, 8_ = 15.2, p < 0.05; *F*_1, 8_ = 3.83, p < 0.1; *F*_1, 8_ = 41.7, p < 0.001; *F*_1,8_ = 14.2, p < 0.05) (Fig 5).

#### Bacterial entomopathogen: *Bacillus thuringiensis*

**Fig 6:**
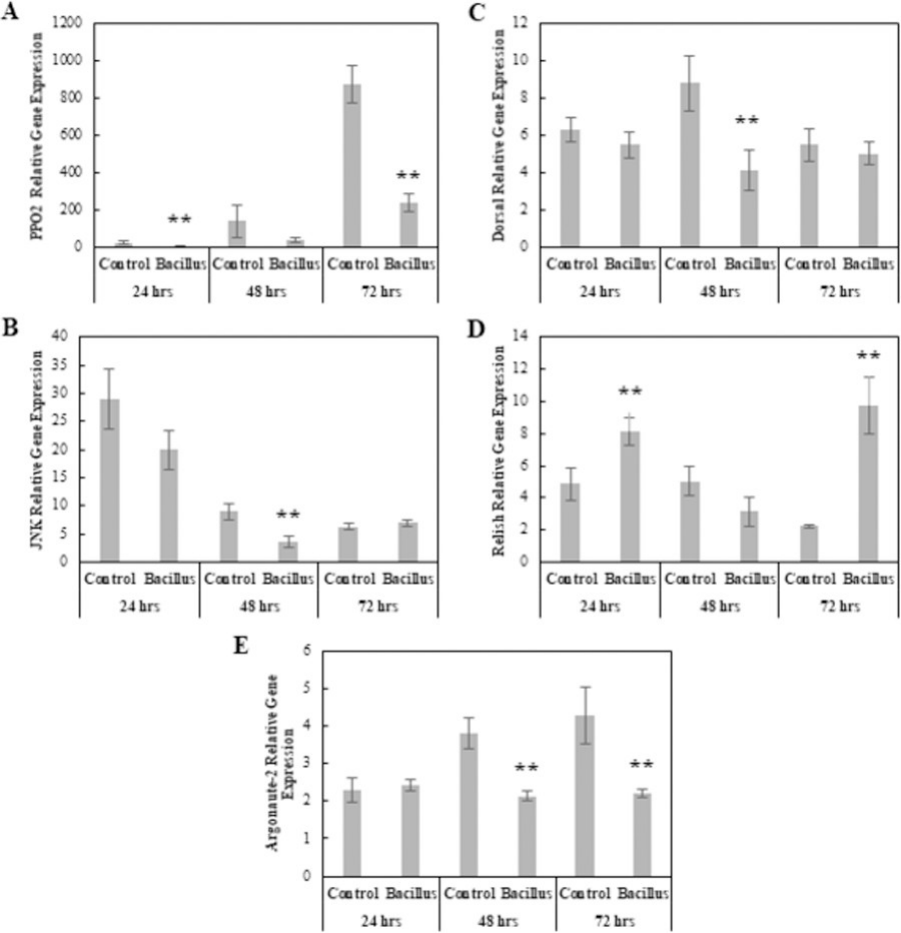
Comparison of relative expression levels of immune related genes in control and *Bacillus thuringiensis*-infected *Helicoverpa zea*. (A) *PPO-2* (Mean ± SE), (B) *JNK* (Mean ± SE), (C) *Dorsal* (Mean ± SE), (D) *Relish* (Mean ± SE), and (E) *Argonaute-2* (Mean ± SE). *Actin* was used as the housekeeping gene. The single asterisk indicates a significant effect (ANOVA, *P* < 0.1) of treatment relative to the Control at that time point. The double asterisk indicates a stronger significant effect (ANOVA, *P* < 0.05) of treatment relative to the Control at that time point (*n* = 5 insects per treatment).

The MANOVA results revealed a significant effect by Treatment (Pillai = 0.719, *F*_5, 20_ = 10.26, p < 0.001), Time (Pillai = 1.54, *F*_10, 42_ = 13.98, p < 0.001), and Treatment × Time interaction (Pillai = 1.09, *F*_10, 42_ = 4.99, p < 0.001). At 24 hpi, ANOVAs revealed a significant increase in *Relish* gene expression levels in *B*. *thuringiensis*-infected larvae compared to Control gene levels (*F*_1, 8_ = 6.15, p < 0.05), and a significant decrease in *PPO-2* expression levels (*F*_1, 8_ = 12.5, p < 0.05), with no differences in expression levels for *Argonaute-2*, *JNK*, or *Dorsal* (Fig 6). At 48 hpi, *Argonaute-2*, *Dorsal*, and *JNK* expression levels were significantly lower in *B*. *thuringiensis*-infected larvae (*F*_1, 8_ = 15.8, p < 0.05; *F*_1, 8_ = 6.5, p < 0.05; *F*_1, 8_ = 9.04, p <0.05), with *Relish* and *PPO-2* expression levels not being significantly different between treatments (Fig 6). By 72 hpi, *Argonaute-2* and *PPO-2* expression levels were significantly reduced in *B*. *thuringiensis*-infected larvae (*F*_1, 8_ = 7.24, p < 0.05; *F*_1, 8_ = 31.3, p < 0.05), and *Relish* expression levels were significantly higher than the Control group (*F*_1, 8_ = 18.6, p < 0.05) (Fig 6).

#### Fungal entomopathogen: *Beauveria bassiana*

**Fig 7:**
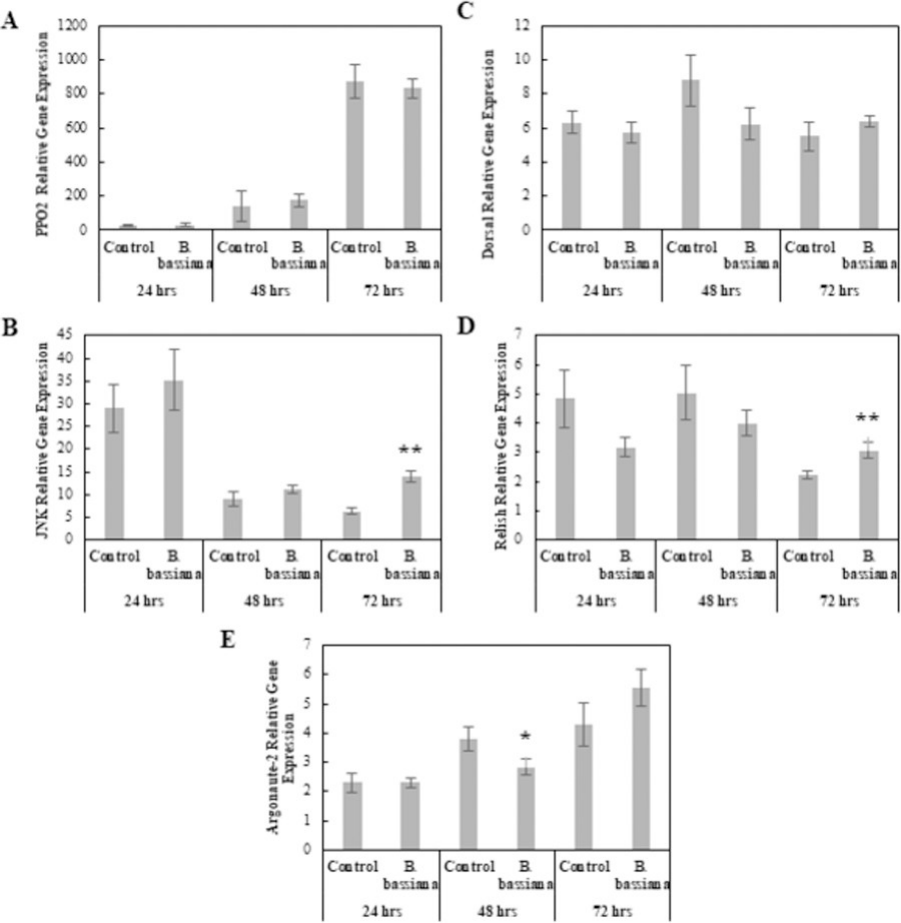
Comparison of relative expression levels of immune related genes in control and *Beauveria bassiana*-infected *Helicoverpa zea*. (A) *PPO-2* (Mean ± SE), (B) *JNK* (Mean ± SE), (C) *Dorsal* (Mean ± SE), (D) *Relish* (Mean ± SE), and (E) *Argonaute-2* (Mean ± SE). *Actin* was used as the housekeeping gene. The single asterisk indicates a significant effect (ANOVA, *P* < 0.1) of treatment relative to the Control at that time point. The double asterisk indicates a stronger significant effect (ANOVA, *P* < 0.05) of treatment relative to the Control at that time point (*n* = 5 insects per treatment).

The MANOVA results showed Time as the only significant main effect (Pillai = 1.61, *F*_10, 42_ = 17.21, p < 0.001), and both Treatment and the Treatment × Time interaction were not significant; therefore, no immune response was observed in *B*. *bassiana*-infected larvae compared to the Control larvae. Although the main effect of Treatment was not significant in the MANOVA, likely due to small sample size, examination of the underlying univariate responses did suggest biologically-relevant changes in gene expression (Fig 7). These changes were reflected in the univariate ANOVAs which indicated that by 48 hpi, *Argonaute-2* expression was significantly reduced compared to Control expression levels (*F*_1, 8_ = 4.14, p < 0.1) (Fig 7). At 72 hpi, *JNK* and *Relish* expression levels were significantly higher than the Control (*F*_1, 8_ = 26.4, p < 0.001; *F*_1, 8_ = 6.8, p < 0.05) (Fig 7).

#### Entomopathogenic nematode: *Steinernema carpocapsae*

**Fig 8:**
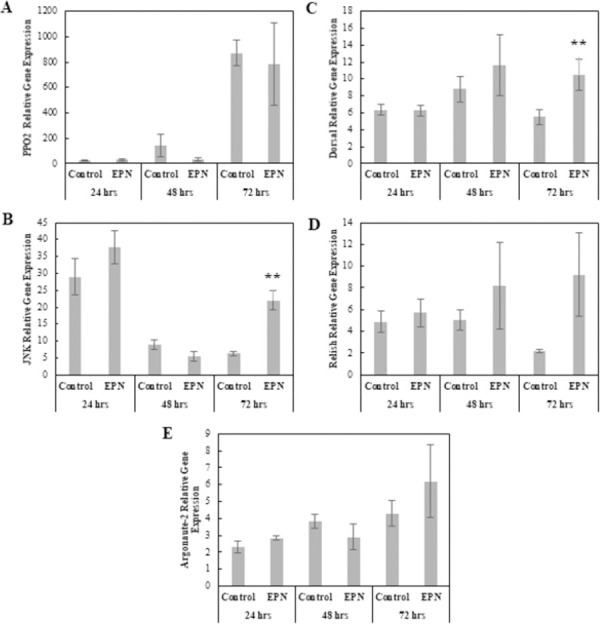
Comparison of relative expression levels of immune related genes in control and *Steinernema carpocapsae*-infected *Helicoverpa zea*. (A) *PPO-2* (Mean ± SE), (B) *JNK* (Mean ± SE), (C) *Dorsal* (Mean ± SE), (D) *Relish* (Mean ± SE), and (E) *Argonaute-2* (Mean ± SE). *Actin* was used as the housekeeping gene. The single asterisk indicates a significant effect (ANOVA, *P* < 0.1) of treatment relative to the Control at that time point. The double asterisk indicates a stronger significant effect (ANOVA, *P* < 0.05) of treatment relative to the Control at that time point (*n* = 5 insects per treatment).

The MANOVA results showed both Treatment and Treatment × Time interaction did not have a significant effect on the data, only Time was a significant effect (Pillai = 1.53, *F*_10, 42_ = 13.82, p < 0.001); therefore, no immune response was detected in *S*. *carpocapsae*-infected larvae compared to Control larvae. Although the main effect of Treatment was not significant in the MANOVA, likely due to small sample size, examination of the underlying univariate responses did suggest biologically-relevant changes in gene expression (Fig 8). These changes were reflected in the univariate ANOVAs which indicated that the 72-hpi samples of *JNK* and *Dorsal* expression levels were significantly elevated compared to the Control treatment (*F*_1, 8_ = 27.2, p < 0.001; *F*_1, 8_ = 5.94, p < 0.05) (Fig 8).

## Discussion

We demonstrate that *Helicoverpa zea* responds differently to each major pathogen group during different stages of infection at a physiological and transcriptional level. Of note, the *H*. *zea* immune response to *Bacillus thuringiensis* was the most robust of all the pathogens we tested. Both PPO and PO concentrations were significantly higher in *B*. *thuringiensis*-infected larvae at 24 and 48 hpi compared to Control larvae. This increase in PPO and PO levels could be an indicator of increased nodule formation as supported by the significant increase in encapsulation ability of infected larvae over the Control and the reduction in hemocytes simultaneously observed at 48 hpi [21]. Interestingly, there were no significant differences in lysozyme-like activity between treatments for any sample point; however, gene expression of *Relish* was significantly higher at 24 and 72 hpi. This increase in expression of the IMD transcription factor should indicate an increase in AMPs with activity against *Bacillus thuringiensis* due to the DAP-type PGNs [78]. Concurrently, there was a significant reduction in *Dorsal* expression at 48 hpi, indicating a reduction in AMPs with activity against most Gram-positive bacteria and fungal pathogens. Furthermore, *Argonaute-2* gene expression was significantly down-regulated compared to the Control at 48 and 72 hpi, possibly due to resource allocation away from antiviral activity. In all, *Bacillus thuringiensis* induced the most robust immune response, which resulted in the up-regulation of *Relish* and the down-regulation of *Dorsal*, and also showed evidence of nodulation formation.

*Helicoverpa zea* larvae infected with HearNPV demonstrated an initial increase in hemocytes at 4 hpi compared to the Control larvae, possibly revealing the importance of hemocytes in an antiviral role as described by Trudeau et al. [26] and McNeil et al. [79, 80]. Unlike *T*. *ni* infected with TnSNPV, total hemocytes peaked early during the infection at 4 hpi rather than 48 hpi as observed by Scholefield et al. [81]. Our physiological data is similar to Pan et al. [82] in that we did not see a prolonged induced response of hemocyte counts or PO concentrations. We also observed an overall decrease in encapsulation ability compared to the Control even when hemocyte counts remained the same. This could be indicative of host hemocytes being exploited and controlled by the viral pathogen prior to lysing and release of viral progeny [83, 84]. Transcriptionally, HearNPV-infected larvae had significantly lower *PPO-2* gene expression compared to the control at 72 hpi, further indicating a lack of viricidal activity, active silencing by HearNPV, or a reduction in healthy cells producing *PPO-2* transcripts. Both *Dorsal* and *Relish* were downregulated compared to the Control, possibly revealing a diversion of resources away from AMP production; however, JNK expression never altered significantly from the Control. This implies that HearNPV infections are not inducing apoptosis via the JNK pathway. Interestingly, *Argonaute-2*, the gene encoding the cleavage protein in the siRNA antiviral pathway was not differentially expressed from the Control until 72 hpi, when it was counterintuitively downregulated, possibly implying a silencing effect by HearNPV, or a reduction in uninfected cells leading to lower overall expression of endogenous genetic material including the gene encoding AGO2. The lack of a substantial immune response by *H*. *zea* to HearNPV infection is likely due to *H*. *zea* being a fully-permissive host of HearNPV, while a semi-permissive or non-permissive host might mount an effective antiviral response [83, 84]. We did not find evidence of effective up-regulation of the siRNA pathway against HearNPV contrary to Jayachandran et al. [29]. This difference in results could be attributed to the differences between utilizing cell lines and whole organism studies. In our study, *Argonaute-2* was not differentially expressed until late in the infection, when it was down-regulated. This down-regulation suggests the potential of HearNPV to silence the siRNA pathway in *H*. *zea*, possibly due to *H*. *zea* being a fully-permissive host [83, 84].

*Beauveria bassiana* did not elicit an immune response in our analyses. All physiological measurements were not significantly different from the Control, except PPO concentrations at 24 hpi which were marginally lower than the Control. These data, coupled with no differences between *B*. *bassiana*-infected and Control larval *PPO-2* expression levels indicate PPO and PO are not important *H*. *zea* immune responses to *B*. *bassiana*. The only genes that were differentially expressed with *B*. *bassiana* infection were *JNK* and *Relish* at 72 hpi, with both being significantly up-regulated compared to the Control. Once again, these data indicate a surprising lack of an observed immune response by *H*. *zea* towards *B*. *bassiana*, even at a transcriptional level, which is startling considering the wide host range *B*. *bassiana* is capable of infecting [85]. It remains to be seen if this lack of an observed response extends to other immune response pathways or other fungal pathogens.

*Steinernema carpocapsae*-infected larvae did not differ from Control larvae in PPO or PO concentrations, or in *PPO-2* expression levels indicating that the PO cascade does not contribute significantly to the immune response of *H*. *zea* to *S*. *carpocapsae* which is surprising considering encapsulation, requiring PO, is the primary immune response to nematode infections [86]. Furthermore, there was a significant reduction in encapsulation ability in infected larvae at 24 hpi, but no difference by 48 hpi. However, there was a significant decrease in hemocytes in infected larvae by 48 hpi. The only two genes differentially expressed were *JNK* and *Dorsal* at 72 hpi. This upregulation and associated physiological response of reduced hemocytes is consistent with the host immune response towards the bacterial symbiont carried by *S*. *carpocapsae*. These data are further evidence of a potential immune-masking ability by the nematode’s cuticle, with little to no evidence of encapsulation occurring, but subsequent up-regulation of genes associated with an immune response against Lys-type gram positive bacteria by 72 hpi [22, 56]. Therefore, *S*. *carpocapsae* did not appear to elicit an immune response; however, the immune response elicited is indicative of a Lys-type gram positive bacterial infection, possibly resulting from septicemia or opportunistic bacteria, as *Xenorhabdus nematophila*, the bacterial symbiont of *S*. *carpocapsae*, is gram negative and has been shown to suppress the host immune response [87].

In conclusion, this study provided a novel assessment of the immune response of a non-model organism at both the physiological and transcriptional levels, to multiple pathogen groups at three time-points during the infection. It provides the foundation for future studies investigating the ecology and evolution of pathogen resistance in *Helicoverpa zea*. Our findings indicate that the *H*. *zea* immune system responds differently depending on the pathogen invading and the specific time course of an infection. We also highlight the lack of importance for the PO cascade in *H*. *zea* immune response to all pathogens utilized except *B*. *thuringiensis*. While this study provides heretofore unreported information about *H*. *zea* immunity, future studies are necessary to explore the differences between semi-permissive and fully-permissive hosts of HearNPV or other viral pathogens, and exploration into differences between cell lines and larval immune assays. Furthermore, insects used in the current study were from a domesticated strain acquired from Benzon Research Inc. (Carlisle, PA). The possibility of wildtype populations exhibiting different immune responses compared to highly domesticated lineages should be explored [88, 89]. Further studies should realize the benefits of utilizing both physiological and transcriptional analyses and implement multiple pathogens and sampling points to gain a clearer picture of how the insect is responding. Furthermore, non-model insect immune assessments are infrequent but necessary to fill key knowledge gaps such as understanding the importance of the PO cascade in immunity. The revelation of a complete lack of observable immune response to *B*. *bassiana* is startling and further promotes the need for studies in non-model organisms.
